# Supplementary material for: A dopaminergic switch for fear to safety transitions
Source: Nat Commun. 2018 Jun 27;9:2483. doi: 10.1038/s41467-018-04784-7 (PMC6021378; doi:10.1038/s41467-018-04784-7)
Supplement: Supplementary file 1 — Supplementary Information [file 41467_2018_4784_MOESM1_ESM.pdf]

Supplementary Information for "A dopaminergic switch for fear to safety transitions", *Luo, Uematsu et al.*

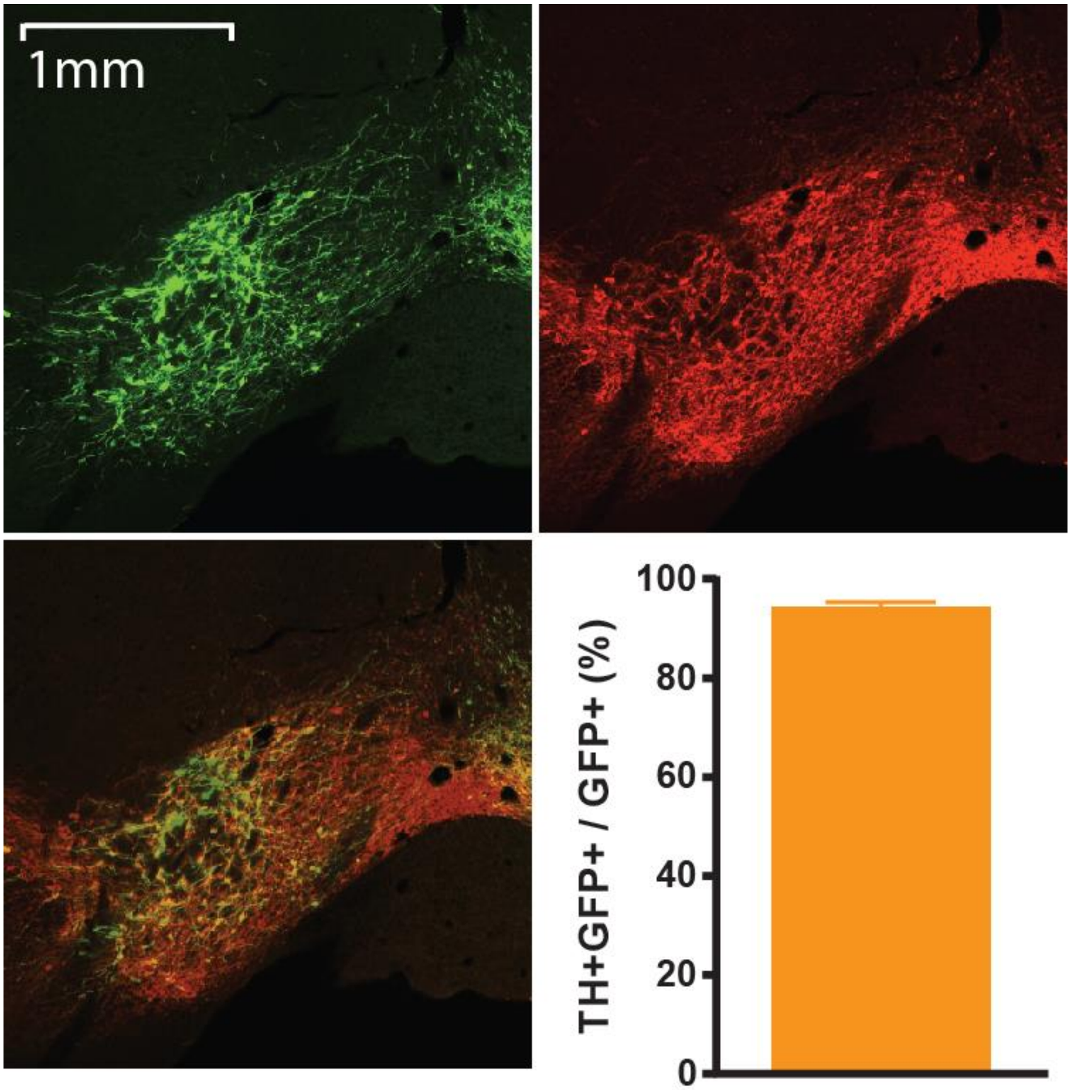

Supplementary Figure 1: Expression in TH-Cre animals is specific to TH+ cells in VTA. GFP expression (green) in TH+ neurons (red) in lateral VTA of TH-Cre rats. Lower left is overlay of green and red channels. Lower right is percentage of GFP+ cells that colabeled for TH+ (n = 7, averages of 4-7 slices/animal, 94.4%). Error bars are S.E.M.

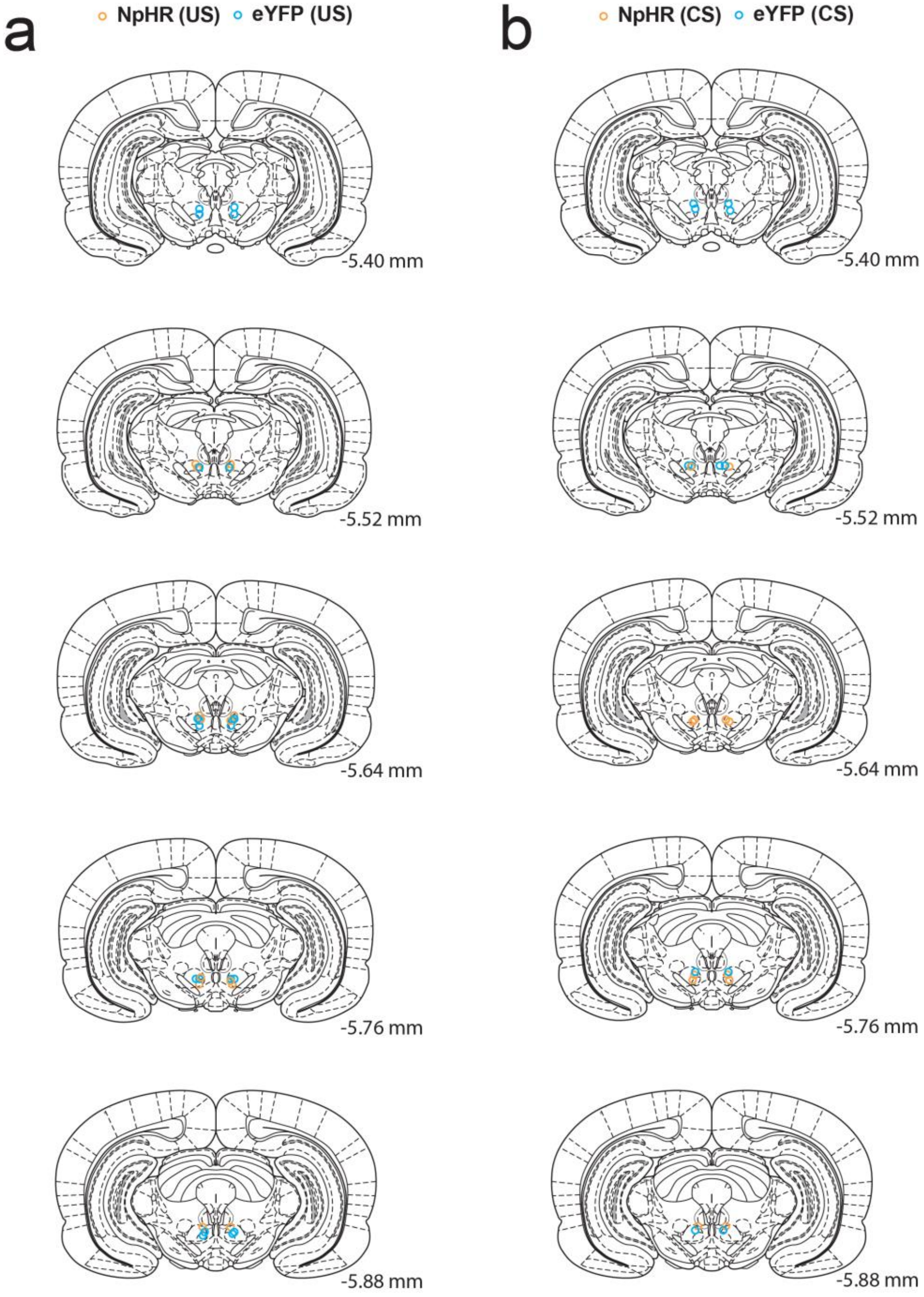

Supplementary Figure 2: Location of fiber implantations obtained from histological reconstruction for VTA dopamine cell inhibition. (**a**) Locations of the tips of optical fibers in VTA for experiments where light inhibition took place during expected omission periods of fear extinction (Fig. 1c). (**b**) Locations of the tips

of optical fibers in VTA for experiments where light inhibition took place during the entire CS periods of extinction training (Fig. 1d). Images were adapted from Ref. [1]. Copyright 1982, Elsevier.

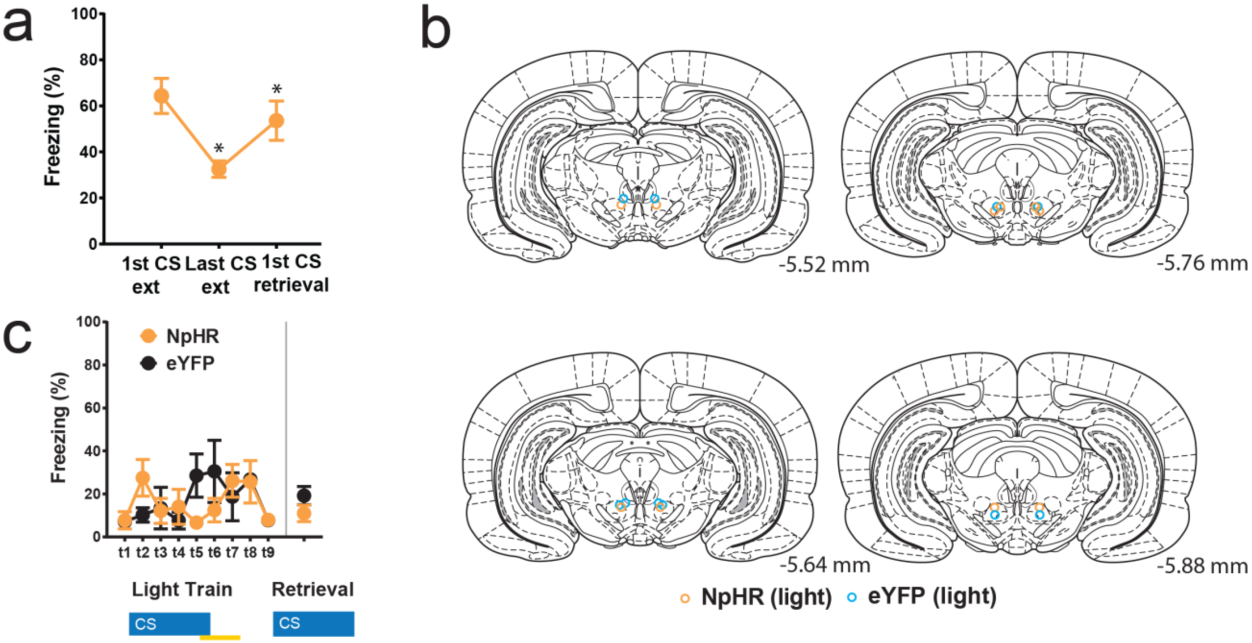

Supplementary Figure 3: Detailed extinction analysis and pairing an auditory CS with optical inhibition of VTA dopamine cells as a US in the absence of shock does not produce fear conditioning. (**a**) In NpHR treated animals which received laser inhibition of VTA dopamine neurons during shock omission (see Fig. 1c), behavioral freezing response (y-axis) during the first auditory CS of fear extinction, last CS of extinction and first CS of the Retrieval test. (n = 8, $F_{2, 18}$ = 5.44, *p* = 0.014, 1 way RM ANOVA) *denote Holm-Sidak post-hoc tests significant difference compared with 1st CS of extinction. (**b**) Locations of optical fiber tips in VTA for experiments for experiments in ***c***. Brain images were adapted from Ref. [1]. Copyright 1982, Elsevier. (**c**) Pairing CS with laser stimulation alone does not produce fear learning. No differences in freezing between NpHR and eYFP groups were evident during training ('Light Train') (n = 5 NpHR, 5 eYFP, 1-trial each, *p* = 0.0844 no interaction, 2-way repeated measures ANOVA) or later when the auditory CS was presented alone (5-trial average, p = 0.1880, Students's t-test). Error bars indicate SEM.

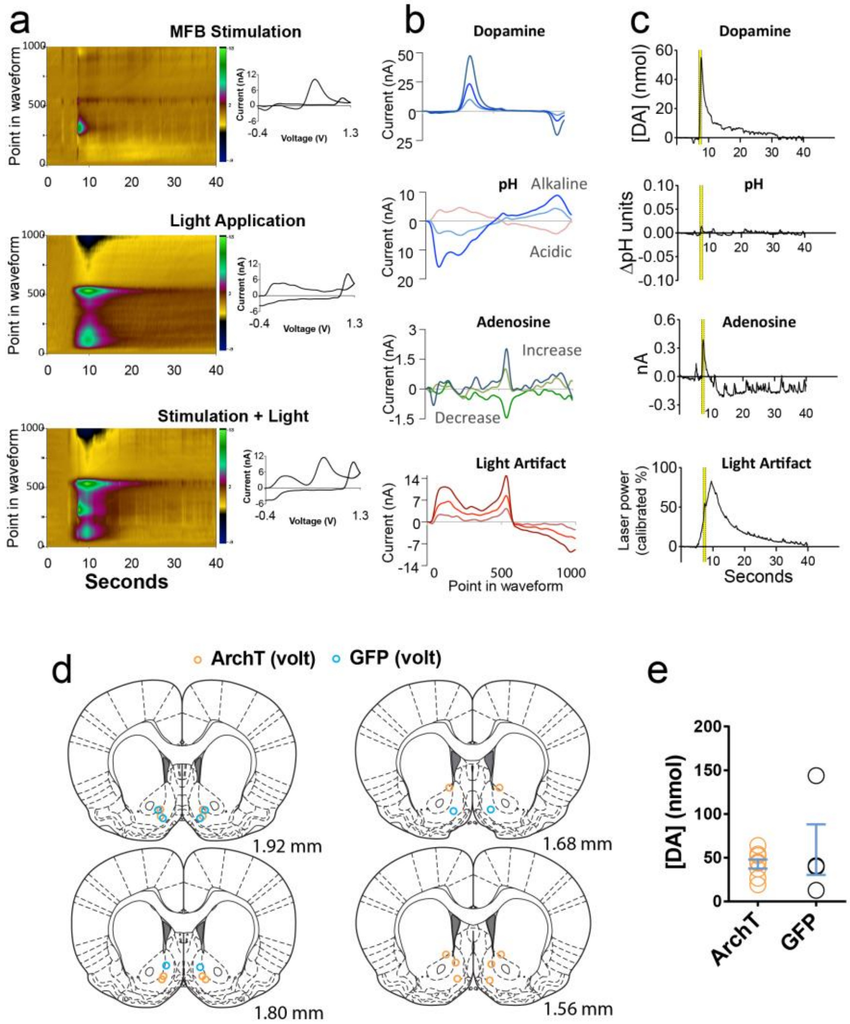

Supplementary Figure 4: Additional voltammetry analyses. Voltammetric signals generated by electrical stimulation and light application. (**a**) Color plots (left) and current-voltage (CV) curves (right) of differential current generated by MFB stimulation (upper panel), light application (middle panel) and simultaneous light and electrical application (lower panel). (**b**) Training templates obtained in vivo for calibration of dopamine, pH shift, adenosine and light artifact. Laser power was applied at graded intensities to obtain graded amplitudes for calibration (represented by shaded colors). (**c**) Separation of all factors by principal component regression analysis of the lower panel (MFB stimulation and light) in ***a***. Yellow shading indicates time of medial forebrain bundle stimulation. (**d**) Locations of optical fiber-carbon fiber bundles (based on lesion marking) in NAc for voltammetry experiments with light illumination (Fig. 3c). Brain images were adapted from Ref. [1]. Copyright 1982, Elsevier. (**e**) Evoked dopamine release without optical inhibition does not differ between ArchT and GFP groups. Dopamine concentrations evoked by MFB

stimulation without laser illumination do not differ between ArchT and GFP injected animals (n = 8 ArchT, 4 GFP, 5-trial average, p = 0.4475, Student's t-test). Error bars indicate S.E.M.

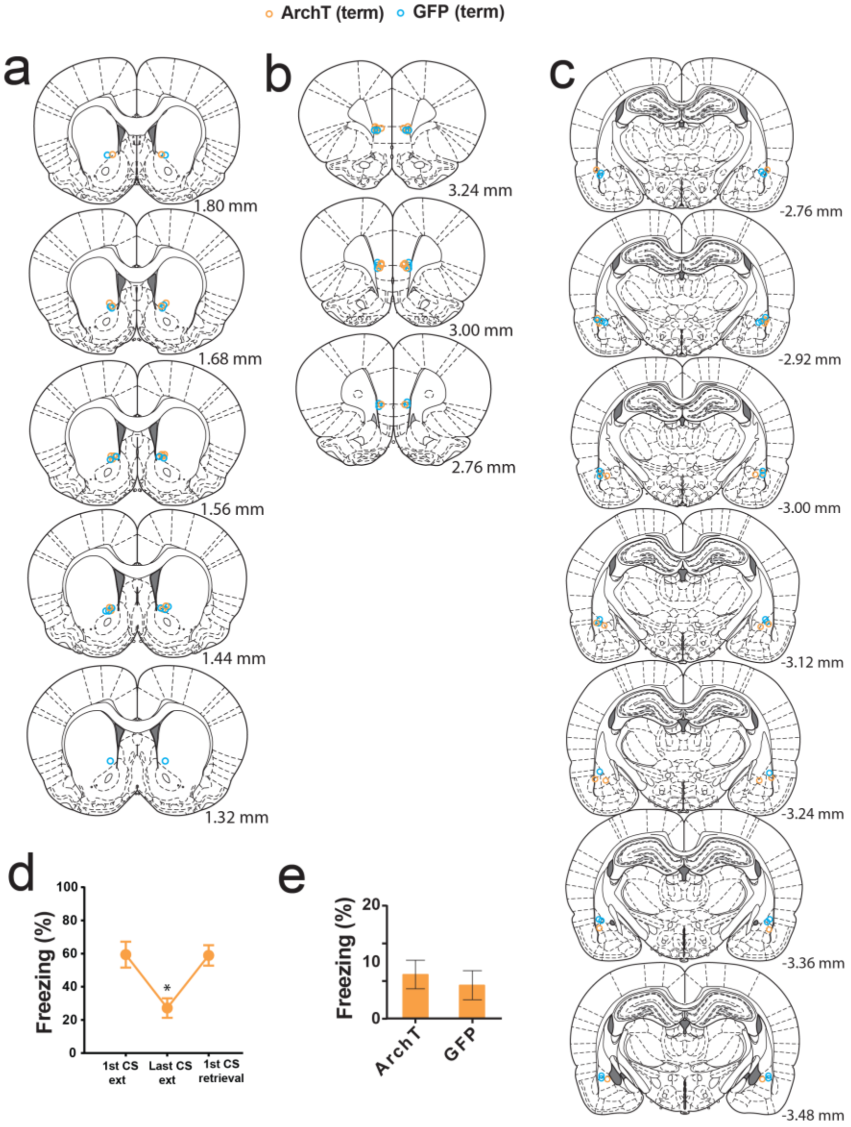

Supplementary Figure 5: Location of optical fibers for terminal manipulation experiments. (**a**) Locations of optical fibers in NAc in experiments involving terminal inhibition during periods of expected shock in fear extinction (Fig 3e). (**b**) Locations of optical fibers in IL in experiments involving terminal inhibition during periods of expected shock in fear extinction (Fig 3g). (**c**) Locations of optical fibers in lateral/basal amygdala in experiments involving terminal inhibition during periods of expected shock in fear extinction (Fig 3i). All brain images were adapted from Ref. [1]. Copyright 1982, Elsevier. (**d**) In NAc terminal inhibition experiment (ArchT treated group) (see Fig. 3e), behavioral freezing response (y-axis) during the first auditory CS of fear extinction, last CS of extinction and first CS of the Retrieval test. (n = 9, $F_{2, 14} = 9.56$, p = 0.0024, 1 way RM ANOVA) *denote Holm-Sidak post-hoc tests significant difference compared with 1st

CS of extinction. (**e**) Baseline freezing levels at Retrieval test for the optical inhibition of VTA-dopamine terminals in NAc experiment (see Fig. 3e) are not different comparing ArchT and GFP treated groups (p=0.609, Student's t-test). Error bars indicate S.E.M.

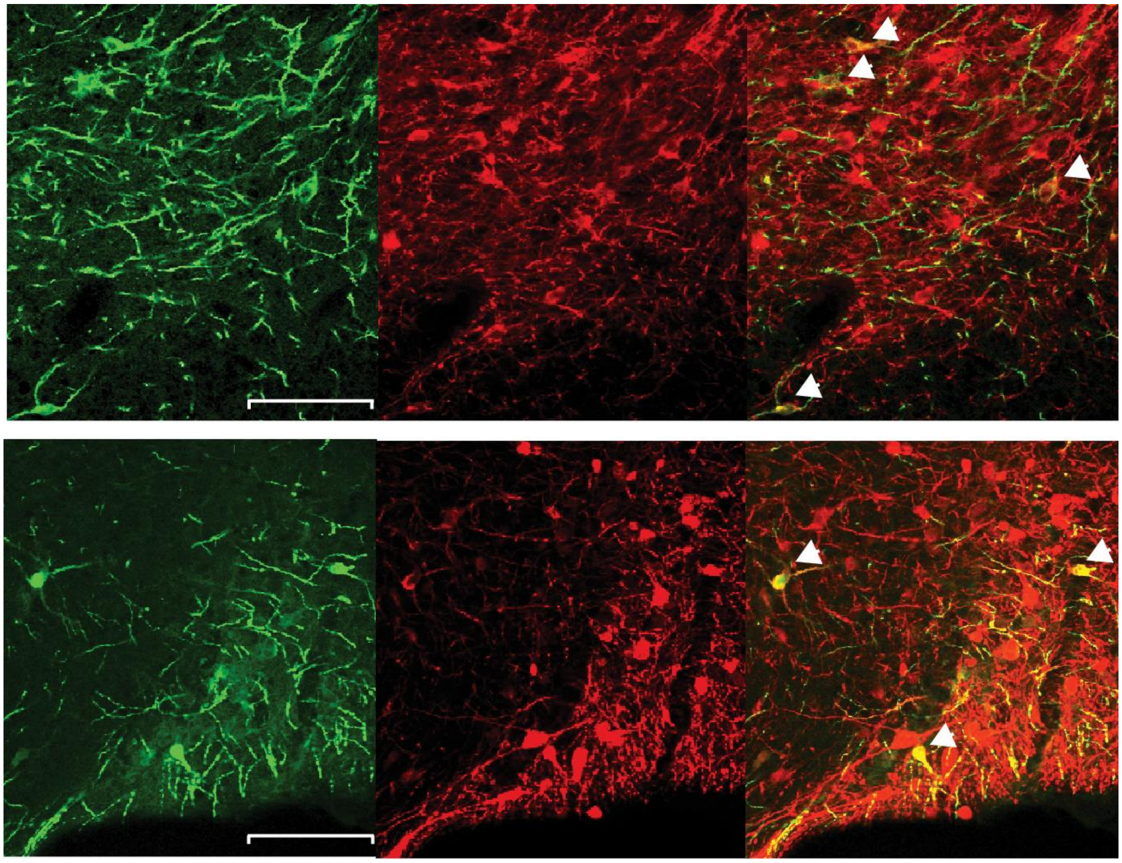

Supplementary Figure 6: Validation of selective opsin infection of VTA-dopamine cells projecting to NAc core or mShell. Examples of combinatorially expressed AAV-fDIO-ArchT3.0-eYFP (green cells, left panels) NAc core (top 3 panels) or mShell (bottom 3 panels) projecting VTA-dopamine neurons (TH stained in red, middle panels) following injection of a retrograde CAV2-FLEX-flp into core or mShell in a TH-cre rat. Right panels show overlays with arrowheads denoting double labelled cells. See Fig. 4b for description of intersectional viral approach.

References

1. Paxinos, G. & Watson, C. *The Rat Brain in Stereotaxic Coordinates*, (Academic Press, New York, 2007).
